# Secondary Metabolites with Antithrombotic and Antioxidant Activities Derived from *Cordyceps cicadae*

**DOI:** 10.3390/molecules31030558

**Published:** 2026-02-05

**Authors:** Xingze Hu, Guisheng Wang, Tao Chen, Xinyue Zhang, Jianying Wu, Guang Shao, Runlin Cai, Zhigang She

**Affiliations:** 1 School of Chemistry, Sun Yat-sen University, Guangzhou 510006, China; huxz@mail2.sysu.edu.cn (X.H.); wanggsh9@mail2.sysu.edu.cn (G.W.); chent296@mail2.sysu.edu.cn (T.C.); wujy89@mail2.sysu.edu.cn (J.W.); shaog@mail.sysu.edu.cn (G.S.); 2 Guangdong Provincial Key Laboratory of Marine Biology, Shantou University, Shantou 515063, China; 23xyzhang1@stu.edu.cn

**Keywords:** *Cordyceps cicadae*, flavonoids, dicarboxylic acid, antithrombotic activity, antioxidant activity

## Abstract

*Cordyceps cicadae*, a medicinal and edible entomopathogenic fungus, has been widely used in traditional Chinese medicine for treating various ailments. This study aimed to validate its ethnopharmacological uses by investigating bioactive constituents and their antithrombotic and antioxidant activities. Through various chromatographic separations, one unreported flavonoid; quercetin-3-*O*-*β*-D-methylglucopyranoside (**1**); three known flavonoids (**2**–**4**); and one new dicarboxylic acid derivative, cicadae acid (**5**), were isolated from *C*. *cicadae*. Their chemical structures were elucidated by a comprehensive spectroscopic analysis (1D/2D NMR and HRESIMS), electronic circular dichroism (ECD) calculations, a DP4+ probability analysis, and the modified Mosher method. All compounds exhibited significant antithrombotic effects at a concentration of 20 μM in a zebrafish model. Compounds **1**–**4** exhibited potent antioxidant activity in the DPPH radical scavenging assay, with IC_50_ values ranging from 12.81 ± 3.42 to 20.16 ± 2.64 μM. These findings provide scientific evidence supporting the traditional application of *C. cicadae*, identifying specific flavonoids and dicarboxylic acids as potential therapeutic agents for thrombosis and oxidative stress-related disorders.

## 1. Introduction

*Cordyceps cicadae* (*C. cicadae*), an entomogenous fungus belonging to the family *Clavicipitaceae* and the genus *Cordyceps*, grows inside the nymph of hosts *Cicadidae* and forms fruiting bodies on the surface of these insects [1,2]. It is primarily distributed in southern China, including Sichuan, Jiangsu, Zhejiang, and Yunnan provinces, where the warm and humid climate is ideal for its growth. Although it has also been reported in Europe, the United States, and Japan, the yields in these regions remain substantially lower than those in China [3]. As a medicinal and edible fungus, *C. cicadae* was documented in ancient pharmacopeias such as the *Compendium of Materia Medica* and the *Formulary of the Taiping People’s Welfare Pharmacy*, as well as in modern authoritative compendiums, including *Chinese Herbal Medicine* and the *Chinese Materia Medica* [4]. For centuries, it has been used as a dietary supplement and folk medicine to treat diabetes, dizziness and chronic kidney diseases [5,6]. Recent studies on *C. cicadae* have identified its primary chemical constituents as nucleosides, sterols, cyclic peptides, polysaccharides, fatty acids and amino acids [7]. These compounds are the material basis for its diverse pharmacological activities [8,9]. For example, adenosine, a major bioactive constituent of *C. cicadae*, has demonstrated therapeutic potential in preclinical studies, particularly for the prevention and management of neurodegenerative disorders [10]. Similarly, ergosterol peroxide, a secondary metabolite isolated from *C. cicadae*, exhibited anti-cancer and renoprotective activities [11]. Furthermore, beauvericin J, which was isolated from *C*. *cicadae*, displayed potent in vitro antitumor activity against hepatocellular carcinoma (HepG2) cells and their multidrug-resistant variant (HepG2/ADM), suggesting potential applications for overcoming chemotherapy resistance [7].

The majority of cardiovascular diseases, such as acute myocardial infarction, ischemic heart disease, and peripheral vascular disease, are associated with thrombotic disorders, contributing to an estimated 20 million deaths each year [12]. The escalating burden of thrombotic disorders is exerting mounting pressure on healthcare systems worldwide [13]. Despite the widespread clinical use of antithrombotics, like acetylsalicylic acid, warfarin, and heparin, their application is limited by adverse effects such as hemorrhage and therapeutic resistance [14,15]. These challenges underscore the critical demand for novel antithrombotic agents with improved safety and efficacy profiles. The zebrafish has emerged as a powerful model organism for screening bioactive compounds due to its unique combination of a high-throughput capability and physiological relevance [16]. This species serves as a critical translational bridge between in vitro assays and mammalian models in modern drug discovery, particularly for thrombosis research [12]. The thrombus formation mechanism in zebrafish closely mirrors human pathophysiology [17]. Importantly, validation studies have confirmed a strong concordance between zebrafish and mammalian responses to clinical antithrombotics, supporting the model’s predictive validity for drug screening [17]. At the molecular level, the zebrafish exhibits an approximate 80% homology with human disease-associated genes, enabling a robust investigation of signalling pathways and targeted drug development [18]. Researchers routinely employ arachidonic acid (AA) to induce thrombus formation, making this model versatile for both high-throughput compound screening and mechanistic studies of antithrombotic agents [19].

Given the extensive ethnopharmacological uses of *C. cicadae* for cardiovascular and metabolic disorders coupled with the urgent need for safer antithrombotic agents, this study systematically investigated its bioactive constituents. Building on prior reports of its nucleosides and sterols (e.g., adenosine and ergosterol peroxide) exhibiting neuroprotective and antitumor properties [7,10,11], the objective of this study was to isolate and characterize previously unexplored secondary metabolites from *Cordyceps cicadae* and to evaluate their antithrombotic and antioxidant activities using a zebrafish thrombosis model. Subsequent studies resulted in the isolation of a new flavonoid, quercetin-3-*O*-*β*-D-methylglucopyranoside (**1**), three known flavonoids (**2**–**4**), and one unreported dicarboxylic acid derivative, cicadae acid (**5**). Herein, the details of the isolation, structural elucidation and biological activities of compounds **1**–**5** are reported.

## 2. Results and Discussion

### 2.1. Structural Elucidation

**Figure 1:**
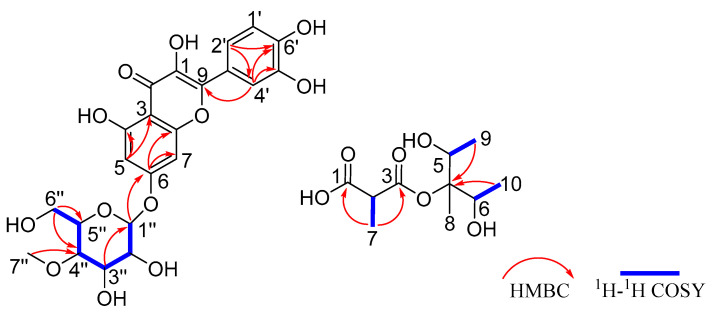
The ^1^H-^1^H COSY (blue bold line) and key HMBC (red arrow) data of compound **1** and **5**.

**Figure 2:**
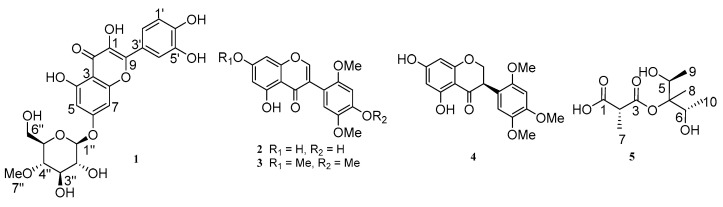
Chemical structures of compounds **1**–**5**.

Compound **1** was obtained as an amorphous yellow oil, and its molecular formula was determined as C_22_H_22_O_12_ based on the HRESIMS protonated ion peak at *m*/*z* 479.1187 [M + H]^+^ (Figure S1). The ^1^H-NMR, ^13^C-NMR and HSQC data (Table 1 and Figures S2–S4) showed that compound **1** had one methyl [*δ*_H_ 3.61 (s, H_3_-7″)], one methylene [*δ*_H_ 3.74 (dd, *J* = 12.2, 4.8 Hz, H-6″a), 3.90 (dd, *J* = 12.2, 4.8 Hz, H-6″b)], five methine [*δ*_H_ 5.05 (d, *J* = 7.7 Hz, H-1″), 3.49 (m, H-2″), 3.63 (d, *J* = 9.1 Hz, H-3″), 3.23 (t, *J* = 9.1 Hz, H-4″), 3.54 (ddd, *J* = 11.0, 5.6, 2.7 Hz, H-6″)], and five aromatic protons [*δ*_H_ 6.45 (d, *J* = 2.2 Hz, H-5), 6.73 (d, *J* = 2.2 Hz, H-7), 7.76 (d, *J* = 2.2 Hz, H-1′), 7.67 (dd, *J* = 8.5, 2.2 Hz, H-2′), *δ*_H_ 6.89 (d, *J* = 8.4 Hz, H-4′)]. The ^13^C-NMR data (Table 1 and Figure S3) displayed a total of 22 carbon signals, including one methyl carbon (*δ*_C_ 60.91), one methylene carbons (*δ*_C_ 62.01), five methine carbons (*δ*_C_ 74.9, 77.4, 77.9, 80.5 and 101.5), five aromatic carbons (*δ*_C_ 95.5, 100.1, 116.1, 116.2 and 121.9) and ten quaternary carbons. The ^1^H-^1^H COSY (Figure 1 and Figure S5) correlations of H-1″/H-2″/H-3″/H-4″/H-6″, together with the HMBC correlations (Figure 1 and Figure S6) from H-6″ to C-4″ and C-5″, from H-3″ to C-1″, and from H_3_-7″ (*δ*_H_ 3.61) to C-4″ confirmed the presence of methylglucoside. In the HMBC spectrum (Figure 1 and Figure S6), the correlations from H-5 to C-3 and C-4, from H-6 to C-7 and C-8, from H-4′ to C-5′, C-6′ and C-9, and from H-2′ to C-4′ and C-6′ confirmed that the aglycone of compound **1** is quercetin [20]. The key HMBC correlation (Figure 1 and Figure S6) from H-1″ to C-6 indicated that methylglucoside was located at the C-6 position. Thus, the planar structure of compound **1** was established and is shown in Figure 2.

Given that the aglycone of compound **1** is quercetin, which is an achiral molecule, the absolute configuration of the entire molecule was determined by the glycosyl unit. H-1″ exhibited a distinct double peak at *δ*_H_ = 5.05, with a coupling constant of *J* = 7.7 Hz in the ^1^H NMR spectrum. This characteristic value corresponds to the *β*-configuration of the glucose pyranose ring [21]. Therefore, the relative configuration can be assigned as *β*. The absolute configuration was determined by an optical rotation analysis. The measured specific rotation for compound **1** was ${[\alpha ]}_{D}^{25}$-24.7 (c 0.1, MeOH). As noted in the work of W. Korytnyk [22] and supported by previous studies [23], it was firmly established that the absolute configuration was the D-configuration. Thus, the compound **1** was confirmed as quercetin-3-*O*-*β*-D-methylglucopyranoside. The known flavonoids were identified as 7-hydroxy-2′,4′,5′-trimethoxyisoflavone (**2**) [24], olibergin A (**3**) [24] and (2*R*)-2,3-dihydro-7-demethylrobustigenin (**4**) [25] by comparing their spectral data with those reported in the literature.

Compound **5** was obtained as an amorphous brown oil, and its molecular formula was determined as C_10_H_18_O_6_ based on the HRESIMS ion peak at *m*/*z* 257.1359 [M + Na]^+^ (Figure S7). The ^1^H-NMR, ^13^C-NMR, and HSQC data (Table 2 and Figures S8–S10) showed that compound **5** had four methyl groups [*δ*_H_ 1.04 (s, H_3_-8), 1.16 (d, *J* = 6.5 Hz, H_3_-10), 1.18 (d, *J* = 6.5 Hz, H_3_-9), 1.36 (d, *J* = 7.0 Hz, H_3_-7)] and three methine groups [*δ*_H_ 3.82 (q, *J* = 6.5 Hz, H-5), 3.84 (q, *J* = 6.5 Hz, H-6), 4.11 (q, *J* = 7.0 Hz, H-2)]. The ^13^C-NMR data (Table 2 and Figure S9) displayed a total of 10 carbon signals, including four methyl carbons (*δ*_C_ 17.5, 17.5, 17.7 and 18.5), three methine carbons (*δ*_C_ 55.5, 72.2 and 72.5) and three quaternary carbons. In the ^1^H-^1^H COSY (Figure S11) correlations between H-2 and H_3_-7 together with the HMBC correlations (Figure S12) from H_3_-7 to C-1 (*δ*_C_ 178.9) and C-3 (*δ*_C_ 159.6), we confirmed the main structure of the malonic acid. The ^1^H-^1^H COSY correlations (Figure 1 and Figure S11) of H-5/H_3_-9 and H-6/H_3_-10, together with the HMBC correlations (Figure 1 and Figure S12) from H_3_-9 to C-4 and C-5, from H_3_-10 to C-4 and C-6, and from H-6 to C-4, confirmed the presence of methylpentanediol on the right side. The chemical shift in C-4 (*δ*_C_ 76.3) and HRESIMS data (Figure S12) confirmed that methylpentanediol was connected to C-3 (*δ*_C_ 159.6) via an oxygen atom. Thus, the planar structure of compound **5** was established and is shown in Figure 2.

**Figure 3:**
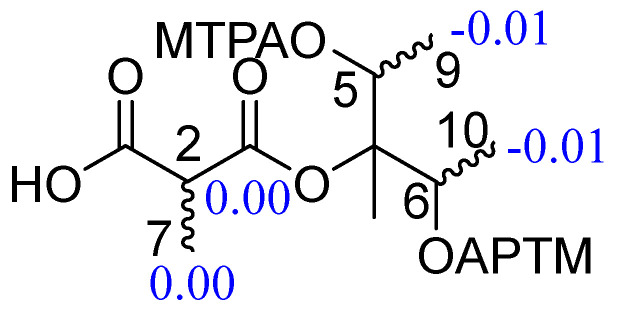
Δ*δ* (=*δS* − *δR*) values for (*S*)- and (*R*)-MTPA esters of compound **5**.

To determine the absolute configuration at C-5 and C-6, the modified Mosher method was used [26]. However, the ^1^H NMR chemical shift differences between the (*R*)-MTPA ester of compound 5 and the (*S*)-MTPA ester of compound 5 were very small (Figure 3, Figures S13 and S14), with a Δ*δ*_H_ (=*δ*_H_*S* − *δ*_H_*R*) of −0.01 at H_3_-9 and H_3_-10. The negligible Δ*δ*_H_ values suggest that the two hydroxyl groups might adopt opposite or quasi-symmetric spatial orientations, resulting in the cancellation of the anisotropic effects of the MTPA moiety and thus nearly unchanged ^1^H chemical shifts [27,28].

**Figure 4:**
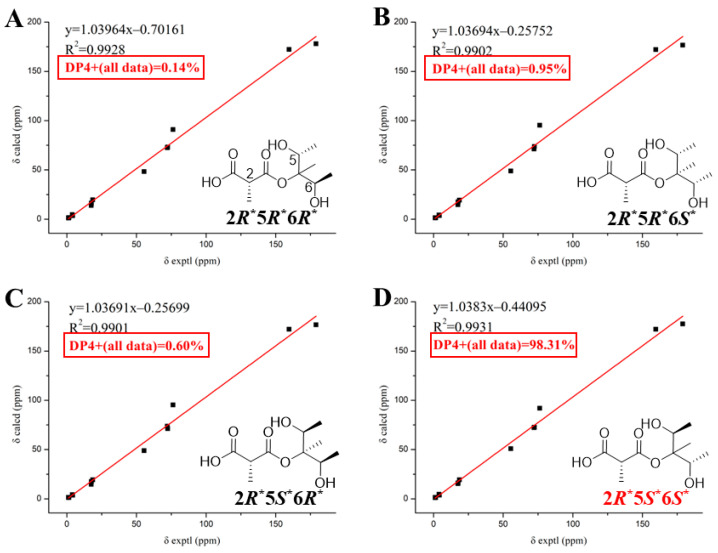
DP4+ analysis of 2R*, 5R*, 6R*-**5** (**A**); 2R*, 5R*, 6S*-**5** (**B**); 2R*, 5S*, 6R*-**5** (**C**); and 2R*, 5S*, 6S*-**5** (**D**) in MeOH.

**Figure 5:**
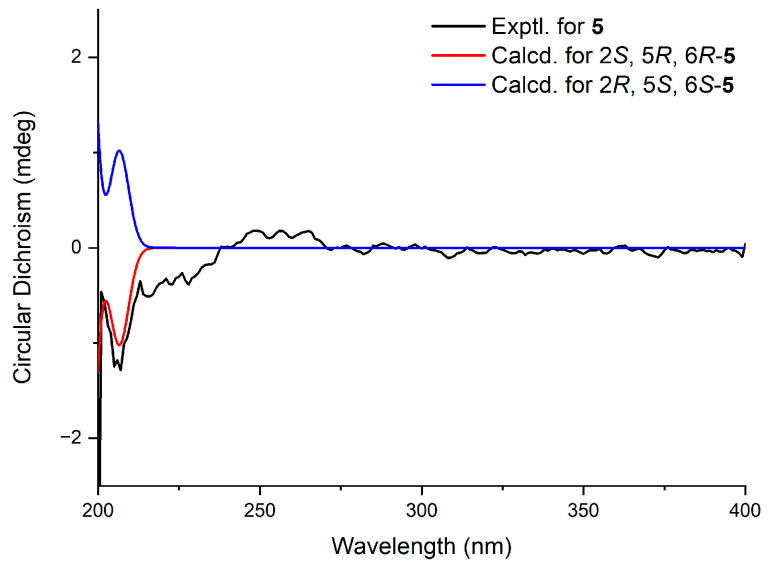
Experimental and calculated ECD spectra of compound **5**.

Compound **5** possesses three stereogenic centres (C2, C5, C6), giving rise to four pairs of relative enantiomeric configurations: 2R*, 5R*, 6R*/2S*, 5S*, and 6S*; 2R*, 5R*, 6S*/2S*, 5S*, and 6R*; 2R*, 5S*, 6R*/2S*, 5R*, and 6S*; and 2R*, 5S*, 6S*/2S*, 5R*, and 6R*. Furthermore, ^13^C NMR calculations were performed for the four possible relative stereochemical configurations of compound **5**: 2R*, 5R*, 6R*-; 2R*, 5R*, 6S*-; 2R*, 5S*, 6R*-; and 2R*, 5S*, 6S*-**5**. The result indicated that 2*R*, 5*S*, 6*S*-**5** was a reasonable structure (Figure 4), with a better correlation coefficient (*R*^2^ = 0.9931) and a high DP4+ probability score at 98.31% (all data) (Figure S15). Eventually, the absolute configuration of compound **5** was established as 2*R*, 5*S*, 6*S* based on ECD calculations (Figure 5).

### 2.2. Antithrombotic Activity in Zebra Fish Model

**Figure 6:**
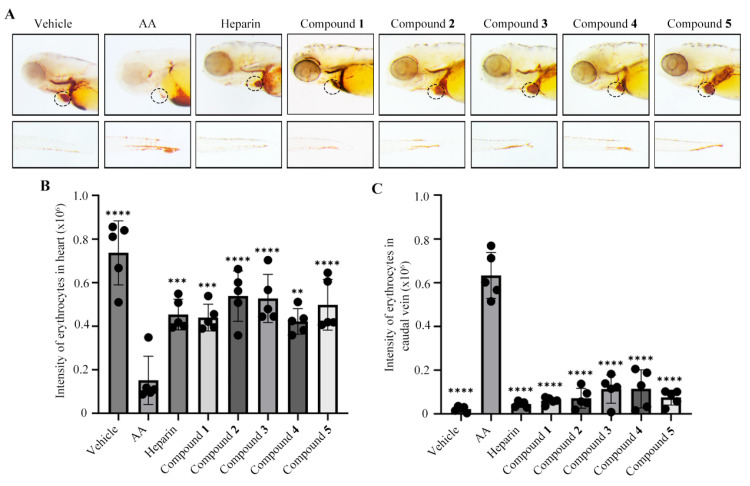
Compounds **1**–**5** (at a concentration of 20 μM) alleviated arachidonic acid (AA)-induced thrombosis in a zebrafish model. Compounds **1**–**5** inhibited thrombosis in zebrafish. (**A**) Typical images of erythrocyte staining both in the heart (**up panel**) and caudal vein (**down panel**). The ellipse indicates the heart area. (**B**,**C**) The quantitative analysis of the intensity of the erythrocyte staining in the heart (**B**) and caudal vein (**C**). The data are shown as the mean ± SD, and Student′ s *t*-test was performed to evaluate the differences between the model group and other groups. ** *p* < 0.01, *** *p* < 0.001, and **** *p* < 0.0001.

The discovery of natural products with antithrombotic activity is of great importance for the development of novel and safer therapeutic agents against thrombotic diseases. Thus, a zebrafish thrombosis model was induced by arachidonic acid (AA) to assess the antithrombotic efficacy [12]. A comparative analysis revealed a significant reduction in the cardiac red blood cell (RBC) staining intensity in the AA model group compared with the control group (Figure 6A,B). This AA-induced decrease was effectively reversed by heparin, which was used as a positive control [29] (Figure 6A,B). The treatment with compounds **1**–**5** at 20 μM significantly restored the cardiac RBC staining intensity, showing effects comparable to those of heparin. In addition, compounds **1**–**5** significantly reduced caudal thrombus areas in zebrafish tails relative to the model group (Figure 6A,C). Taken together, these results indicate that compounds **1**–**5** exhibit antithrombotic activity in the AA-induced zebrafish thrombosis model.

### 2.3. Antioxidant Activity In Vitro

**Figure 7:**
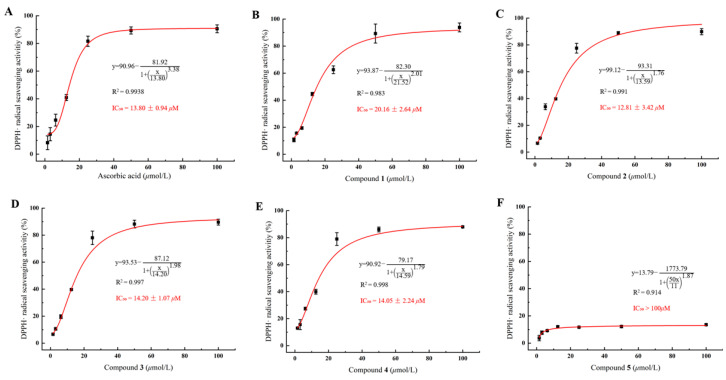
In vitro DPPH· radical scavenging activities of ascorbic acid and compounds **1**–**5** (**A**–**F**). Data are expressed as the mean ± standard deviation (SD) from three independent experiments (*n* = 3).

The DPPH·free radical scavenging assay is a fundamental method for evaluating antioxidant activity in vitro; this method has been widely used for the quantitative determination of the antioxidant capacity of biological samples, phenolic substances, and food [30]. As depicted in Figure 7, compounds **1**–**4** exhibited antioxidant activities comparable to that of the positive control, ascorbic acid (IC_50_ = 13.8 ± 0.94 μM), with IC_50_ values of 20.16 ± 2.64, 12.81 ± 3.42, 14.20 ± 1.07, and 14.05 ± 2.24 μM, respectively. Compound **5** showed weak antioxidant activity, with an IC_50_ value exceeding 100 μM.

## 3. Materials and Methods

### 3.1. General Experimental Procedures

HRESIMS data were recorded using a Thermo-Fisher LTQ-Orbitrap-LC-MS spectrometer (Thermo Fisher Scientific, Palo Alto, CA, USA). The 1D and 2D NMR data were recorded on a Bruker Avance 600 MHz spectrometer (Bruker, Karlsruhe, Germany) at room temperature, utilizing residual solvent signals for calibration (MeOD *δ*_H_/*δ*_C_ 3.31/49.7; CDCl_3_: *δ*_H_/*δ*_C_ 7.26/77.1). Optical rotations were acquired on an MCP300 polarimeterd (Anton Paar GmbH*,* Graz, Austria). Silica gel (200–300 mesh, Qingdao Marine Chemical Factory, Qingdao, China) and Sephadex LH-20 (Amersham Pharmacia, Stockholm, Sweden) were employed as stationary phases for column chromatographic purification. Compounds **1**–**5** were purified by semi-preparative HPLC on a Thermo Scientific Ultimate 3000 BioRS platform (Thermo Fisher Scientific, Germering, Germany) fitted with a Chiralcel AY-H column (5 μm, 4.6 × 250 mm; Daicel Chemical Industries, Tokyo*,* Japan).

### 3.2. Extraction and Isolation

The *C. cicadae* strain used in this study was sourced from Guangzhou Jinchanhua Technology Co. Ltd. (Guangzhou, China). After appropriate cultivation and grinding, a total of 15 kg of powder was obtained for subsequent research. The powder was divided into two 20 L glass vessels. Each portion was extracted successively with MeOH twice, CH_2_Cl_2_:MeOH (1:1, *v*/*v*) once, and *n*-BuOH once. Each extraction lasted for one week. All resulting solutions were combined, concentrated under reduced pressure, and then partitioned with ethyl acetate (EtOAc). The EtOAc phase was concentrated to yield the crude extract. The crude extracts were separated using gradient elution with a petroleum ether/EtOAc mixture (from 9:1 to 0:1, *v*/*v*) on silica gel column chromatography, resulting in 15 fractions (Fr.1–Fr.15). Fr.10 (1200 mg) was further purified on Sephadex LH-20 with a CH_2_Cl_2_/MeOH (1:1, *v*/*v*) solvent system, yielding fractions Fr.10.1 to Fr.10.10. From Fr.10.5 (150 mg), compounds **2** (4.8 mg), **3** (4.3 mg), and **4** (5.1 mg) were isolated after further purification using silica gel with a gradient of CH_2_Cl_2_/MeOH (200:1 to 50:1). Fr.12 (380 mg) was subjected to silica gel column chromatography with a CH_2_Cl_2_/MeOH gradient (100:1 to 20:1), resulting in fractions Fr.12.1–Fr.12.8. Fr.12.8 (30 mg) was then separated on Sephadex LH-20 (Amersham Pharmacia, Stockholm, Sweden) using CH_2_Cl_2_/MeOH (1:1), yielding compound **1** (7.8 mg) and compound **5** (4.2 mg).

Quercetin-3-*O*-*β*-D-methylglucopyranoside (**1**): Amorphous yellow oil; ${[\alpha ]}_{D}^{25}$-24.7 (c 0.1, MeOH); UV (c 0.1, MeOH) *λ*_max_ (log *ɛ*): 370 nm (+1.83), 256 nm (+1.56); IR (KBr) *ν*_max_: 3412, 1608, 1520, 1311, 1242, 1026, 821 cm^−1^; HRESIMS: *m*/*z* 479.1187 [M + H]^+^ (calcd: C_22_H_23_O_12_, 479.1184); ^1^H-NMR (MeOD-*d*_4_, 600 MHz); and ^13^C-NMR (MeOD-*d*_4_, 150 MHz) in Table 1.

Cicadae acid (**5**): Amorphous brown oil; ${[\alpha ]}_{D}^{25}$-14.5 (c 0.1, MeOH); UV (c 0.1, MeOH) *λ*_max_ (log *ɛ*): 210 nm (+2.23); IR (KBr) *ν*_max_: 3412, 3256, 2825, 1735, 1710 cm^−1^; HRESIMS: *m*/*z* 257.1358 [M + Na]^+^ (calcd: C_10_H_18_O_6_, 257.1354); ^1^H-NMR (MeOD-*d*_4_, 600 MHz); and ^13^C-NMR (MeOD-*d*_4_, 150 MHz) in Table 2.

### 3.3. Antithrombotic Activity

#### 3.3.1. Chemical Treatment

The wild-type AB strain zebrafish was utilized in this study. Embryos were cultured in Holt buffer (3.5 g NaCl, 0.05 g KCl, 0.025 g NaHCO_3_, 0.1 g CaCl_2_ per litre of distilled water) and maintained in an incubator at 28.5 °C. At 72 h post-fertilization (hpf), embryos were selected and distributed into 12-well plates, with 20 embryos per well. They were then randomly allocated into four experimental groups: a blank control group (Holt buffer only), a thrombosis model group (80 μM AA, Aladdin, Shanghai, China; A295131), a positive control group (80 μM AA + 100 U/mL heparin), and chemical treatment groups (80 μM AA + 20 μM compounds **1**–**5**). Embryos were pre-incubated with heparin or compounds **1**–**5** for 6 h in the positive control and chemical-treated groups, respectively, whereas the blank control and model groups received Holt buffer instead. Following this incubation, the solution was carefully removed, and all embryos were washed twice with Holt buffer. Subsequently, all groups except the blank control were exposed to 80 μM AA for 1 h in the dark to induce thrombosis. The entire experiment was independently replicated three times.

#### 3.3.2. Erythrocyte Staining and Intensity Analysis

Following AA treatment, zebrafish embryos were subjected to O-dianisidine staining to visualize erythrocyte distribution, as previously described [31]. Stained embryos were randomly selected from each group and imaged using a microscope with Axiocam ERc 5s (Zeiss, Jena, Germany). The staining intensity of erythrocytes in both the heart and caudal vein was subsequently quantified using ImageJ software (version 1.53k, National Institutes of Health, Bethesda, MD, USA).

#### 3.3.3. Statistical Analysis

Statistical analysis was performed using Graphpad Prism 8.3.0 software. Data were presented as the mean ± SD, and differences between the model group and other groups were determined by Student′ s *t*-test. A *p*-value of less than 0.05 was defined as statistically significant.

### 3.4. Antioxidant Activity Assays In Vitro

The DPPH· radical scavenging activities of compounds **1**–**5** were evaluated following a literature method using a 96-well microplate assay [32]. Test samples (1.5625–100 μM, 100 μL) were mixed with DPPH· solution in MeOH (0.16 mM, 100 μL), with ascorbic acid employed as the positive control. After incubation in the dark for 30 min, absorbance was recorded at 517 nm, and the scavenging activity was calculated accordingly.

DPPH· radical scavenging activity (%) = [(Abs_control_ − Abs_sample_)/(Abs_control_ − Abs_blank_)] × 100%

### 3.5. Preparation of MTPA Esters of Compound **5** by the Modified Mosher Ester Method

Compound **5** (1.0 mg) was derivatized with (*S*)-α-methoxy-α-(trifluoromethyl) phenylacetyl chloride [(*S*)-MTPA-Cl, 50 μL] in pyridine-*d*_5_ (500 μL) at room temperature for 12 h, affording the corresponding (S)-MTPA ester (**5a**, 0.853 mg, 85.3% yield). Under identical conditions, treatment with (*R*)-MTPA-Cl produced the (*R*)-MTPA ester (**5b**, 0.842 mg, 84.2% yield). The ^1^H NMR spectra of the (*R*)- and (*S*)-MTPA esters (Figures S13 and S14) were recorded on a Bruker Avance 600 MHz spectrometer (Bruker, Karlsruhe, Germany) at ambient temperature. Proton resonances were assigned on the basis of the ^1^H NMR data and used for calculation of the Δ*δ S−R* values [33].

(*S*)-MTPA ester for **5a**: ^1^H NMR (pyridine-*d*_5_, 600 MHz) *δ*_H_ 4.31 (H-2), 3.65 (H-5), 3.64 (H-5), 1.47 (H_3_-7), 1.26 (H_3_-8), 1.46 (H_3_-9), 1.45 (H_3_-10).

(*R*)-MTPA ester for **5b**: ^1^H NMR (pyridine-d5, 600 MHz) *δ*_H_ 4.31 (H-2), 3.65 (H-5), 3.64 (H-5), 1.47 (H_3_-7), 1.26 (H_3_-8), 1.45 (H_3_-9), 1.44 (H_3_-10).

### 3.6. ECD and ^13^C NMR Calculation Computation Methods

Compound conformers were generated using Spartan 14 (Wavefunction, Irvine, CA, USA), and those with Boltzmann populations >1% were optimized at the B3LYP/6-31+G(d,p) level in methanol using DFT in Gaussian 09. The resulting ^13^*C* NMR and ECD spectra were simulated and processed with SpecDis 1.6 (University of Würzburg, Würzburg, Germany) [34].

## 4. Conclusions

In summary, one previously undescribed flavonoid, 3-*O*-*β*-D-methylglucopyranoside (**1**); three known flavonoids (**2**–**4**); and one new dicarboxylic acid derivative, cicadae acid (**5**), were isolated from the fungus *C. cicadae*. In the bioactivity assays, compounds **1**–**5** exhibited potent antithrombotic activity at a concentration of 20 μM in the zebrafish thrombosis model, marking the first documentation of such activity for flavonoids derived from *C. cicadae*. In addition, compounds **1**–**4** demonstrated significant antioxidant activity in the DPPH· radical scavenging assay, with IC_50_ values ranging from 12.81 ± 3.42 to 20.16 ± 2.84 μM. These findings expanded the chemical diversity of *C. cicadae* and confirmed its potential as a valuable source for bioactive compounds with dual antioxidant and antithrombotic activities. Moreover, this research confirmed that *C. cicadae* could be used as a dietary and health supplement.

## Figures and Tables

**Table 1:** ^1^H (600 MHz) and ^13^C (150 MHz) NMR data of compound **1** in CD_3_OD.

No.	Compound 1	
	*δ*_C_, Type	*δ*_H_, Mult. (*J* in Hz)
C-1	137.6, C	
C-2	177.5, C	
C-3	106.3, C	
C-4	162.2, C	
C-5	100.1, CH	6.45, d (2.2)
C-6	164.4, C	
C-7	95.5, CH	6.73, d (2.2)
C-8	157.7, C	
C-9	146.1, C	
C-1′	116.2, CH	6.89, d (8.5)
C-2′	121.9, CH	7.67, dd (8.5, 2.2)
C-3′	123.9, C	
C-4′	116.1, CH	7.76, d (2.2)
C-5′	148.8, C	
C-6′	148.9, C	
C-1″	101.5, CH	5.05, d (7.7)
C-2″	74.9, CH	3.49, m
C-3″	77.9, CH	3.63, d (9.1)
C-4″	80.5, CH	3.23, t (9.1)
C-5″	77.4, CH	3.54, ddd (11.0, 5.6, 2.7)
C-6″	62.0, CH_2_	3.74, dd (12.2, 4.8)
		3.90, dd (12.2, 4.8)
C-7″	60.9, CH_3_	3.61, s

**Table 2:** ^1^H (600 MHz) and ^13^C (150 MHz) NMR data of compound **5** in CD_3_OD.

No.	Compound 5	
	*δ*_C_, Type	*δ*_H_, Mult. (*J* in Hz)
C-1	178.93, C	
C-2	55.5, CH	4.11, q, (7.0)
C-3	159.61, C	
C-4	76.26, C	
C-5	72.15, CH	3.82, q, (6.5)
C-6	72.46, CH	3.84, q, (6.5)
C-7	17.45, CH_3_	1.36, d, (7.0)
C-8	18.49, CH_3_	1.04, s
C-9	17.52, CH_3_	1.18, d, (6.5)
C-10	17.66, CH_3_	1.16, d (6.5)

## Data Availability

The original contributions presented in this study are included in the article/Supplementary Material. Further inquiries can be directed to the corresponding authors.

## Supplementary Materials

The following supporting information can be downloaded at https://www.mdpi.com/article/10.3390/molecules31030558/s1: Figure S1: HRESIMS spectrum of **1**; Figure S2: ^1^H NMR spectrum of **1**; Figure S3: ^13^C NMR spectrum of **1**; Figure S4: HSQC spectrum of **1**; Figure S5: ^1^H-^1^H COSY spectrum of **1**; Figure S6: HMBC spectrum of **1**; Figure S7: HRESIMS spectrum of **5**; Figure S8: ^1^H NMR spectrum of **5**; Figure S9: ^13^C NMR spectrum of **5**; Figure S10: HSQC^1^ spectrum of **5**; Figure S11: H-^1^H COSY spectrum of **5**; Figure S12: HMBC spectrum of **5**; Figure S13: ^1^H NMR spectrum of (R)-MTPA ester of **5**; Figure S14: ^1^H NMR spectrum of (S)-MTPA ester of **5**; and Figure S15: DP4+ analysis of compound 2*R**5*R**6*R**-, 2*R**5*R**6*S**-, 2*R**5*S**6*R**-, and 2*R**5*S**6*S**-**5**. References [24,25] are cited in the supplementary materials.
