# Supplementary material for: Secondary Metabolites with Antithrombotic and Antioxidant Activities Derived from Cordyceps cicadae
Source: Molecules. 2026 Feb 5;31(3):558. doi: 10.3390/molecules31030558 (PMC12899984; doi:10.3390/molecules31030558)
Supplement: Supplementary file 1 [file molecules-31-00558-s001.zip › molecules-4086092-supplementary.pdf]

Article

Secondary Metabolites with Antithrombotic and Antioxidant Activities Derived from *Cordyceps cicadae*

Xingze Hu [1,†], Guisheng Wang [1,†], Tao Chen [1], Xinyue Zhang [2], Jianying Wu [1], Guang Shao [1], Runlin Cai [2,*] and Zhigang She [1,*]

[1] School of Chemistry, Sun Yat-sen University, Guangzhou 510006, China; huxz@mail2.sysu.edu.cn (X.H.); wanggsh9@mail2.sysu.edu.cn (G.W.); chent296@mail2.sysu.edu.cn (T.C.); wujy89@mail2.sysu.edu.cn (J.W.); shaog@mail.sysu.edu.cn (G.S.)
[2] Guangdong Provincial Key Laboratory of Marine Biology, Shantou University, Shantou 515063, China; 23xyzhang1@stu.edu.cn
* Correspondence: rlcai@stu.edu.cn (R.C.); cesshzhg@mail.sysu.edu.cn (Z.S.)
† These authors contributed equally to this work.

Content

Figure S1 HRESIMS of compound **1.** 3
Figure S2 ^{1}H NMR spectrum of compound **1** in MeOD. 3
Figure S3 ^{13}C spectrum of compound **1** in MeOD. 4
Figure S4 HSQC spectrum of compound **1** in MeOD 4
Figure S5 ^{1}H-^{1}H COSY spectrum of compound **1** in MeOD 5
Figure S6 HMBC spectrum of compound **1** in MeOD 5
Figure S7 HRESIMS of compound **5.** 6
Figure S8 ^{1}H NMR spectrum of compound **5** in MeOD. 6
Figure S9 ^{13}C NMR spectrum of compound **5** in MeOD 7
Figure S10 HSQC spectrum of compound **5** in MeOD. 7
Figure S11 ^{1}H-^{1}H COSY spectrum of compound **5** in MeOD. 8
Figure S12 HMBC spectrum of compound **5** in MeOD. 8
Figure S13. ^{1}H NMR spectrum of (*R*)-MTPA ester of **5**. 9
Figure S14. ^{1}H NMR spectrum of (*S*)-MTPA ester of **5**. 9
Figure S15. DP4+ analysis of compound 2*R**5*R**6*R**-, 2*R**5*R**6*S**-, 2*R**5*S**6*R**-, and 2*R**5*S**6*S**-**5**. 10
Known compounds 2-4 NMR and ESI-MS data: 10
References 11

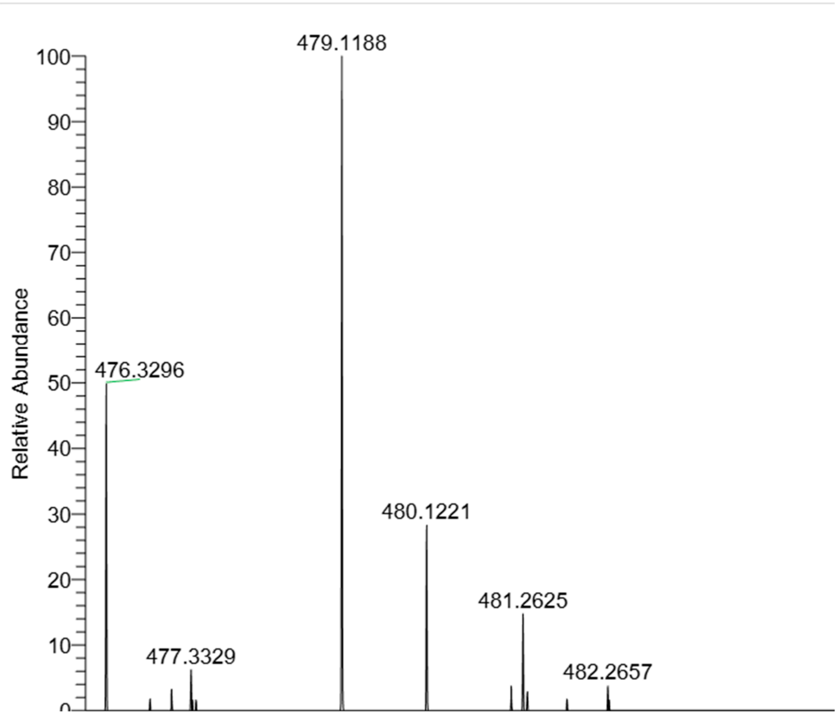

Figure S1. HRESIMS of compound **1.**

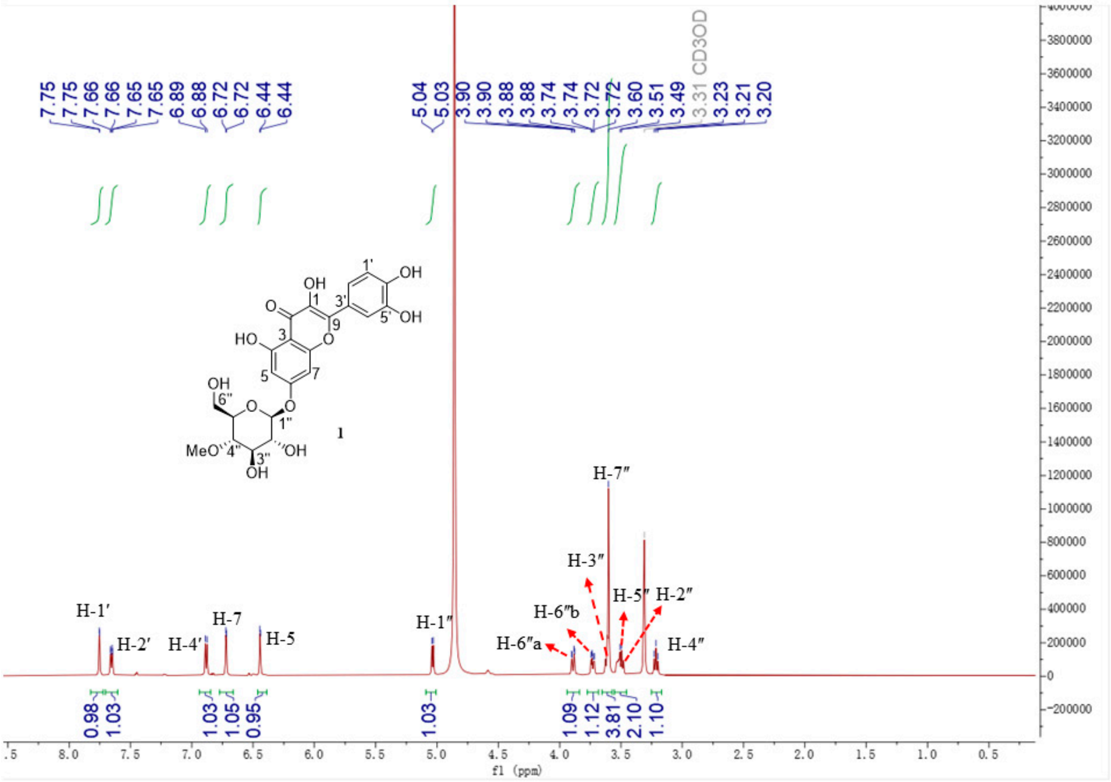

Figure S2. ^{1}H NMR spectrum of compound **1** in MeOD.

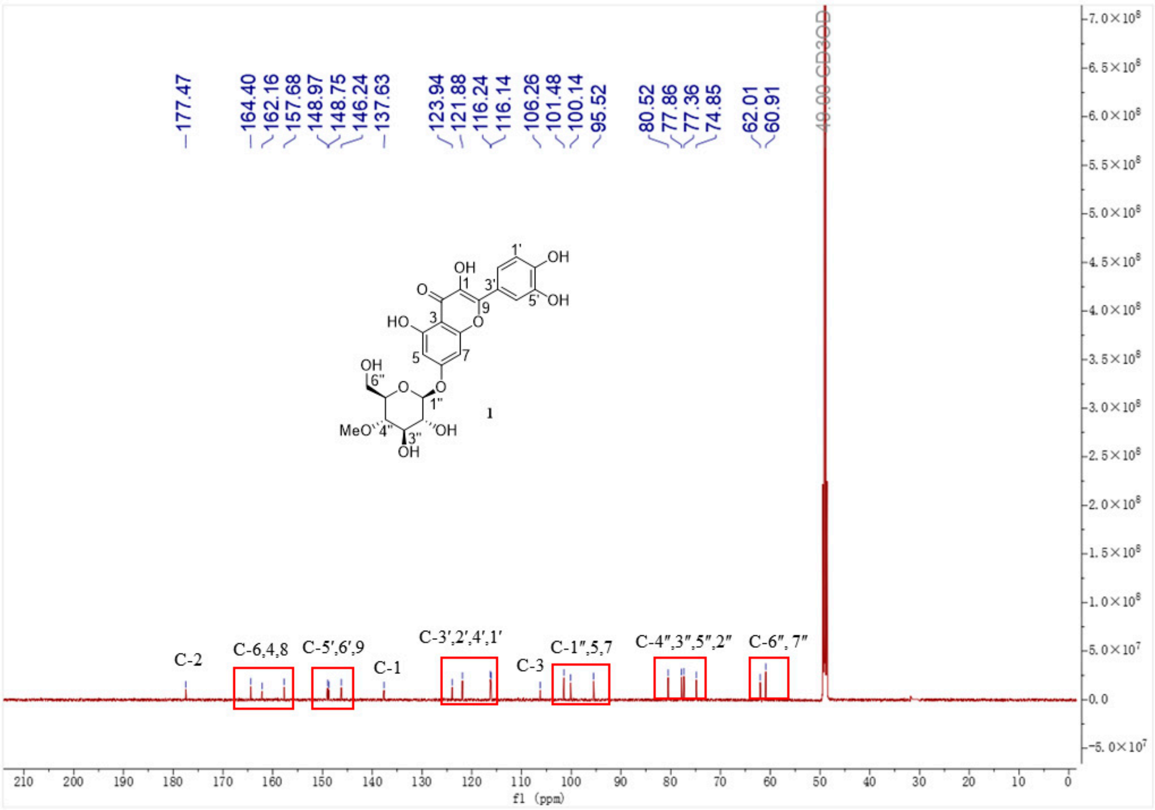

Figure S3. ^{13}C spectrum of compound **1** in MeOD.

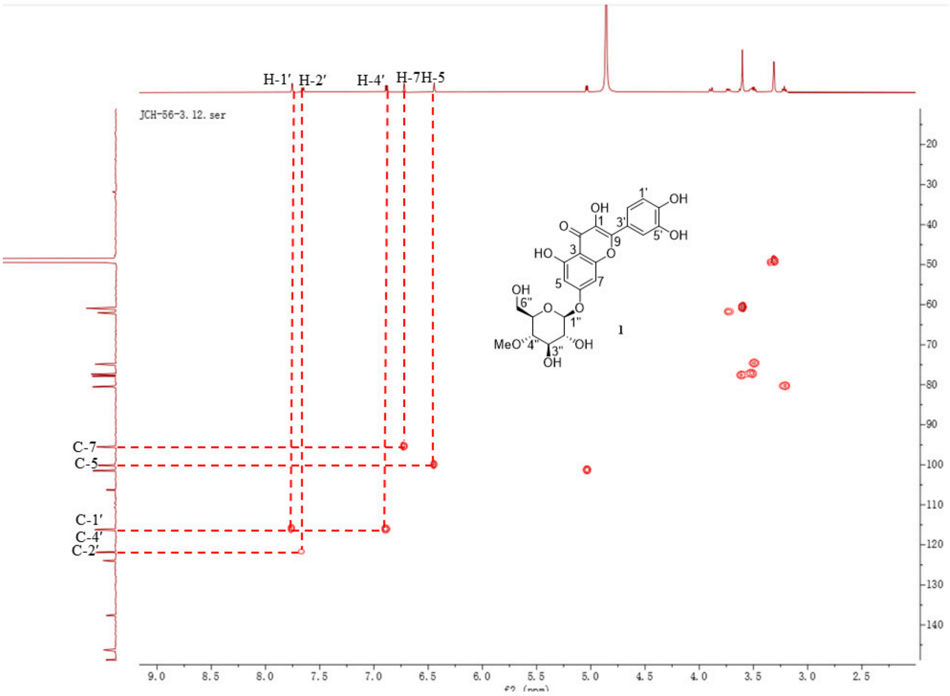

Figure S4. HSQC spectrum of compound **1** in MeOD

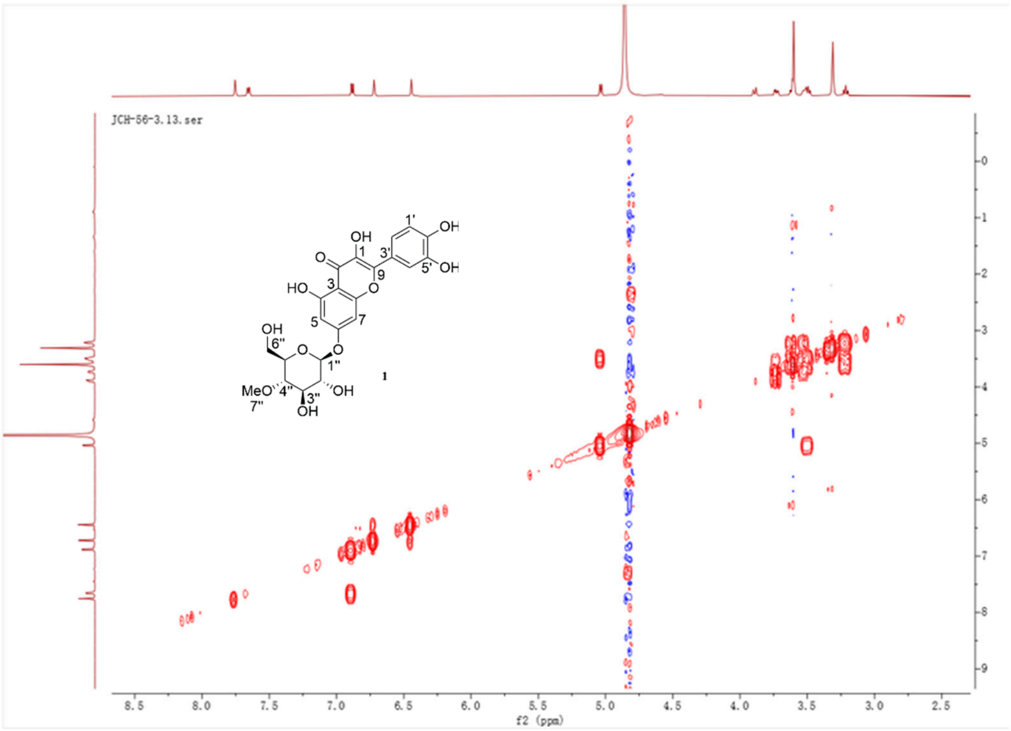

Figure S5. ^{1}H-^{1}H COSY spectrum of compound **1** in MeOD

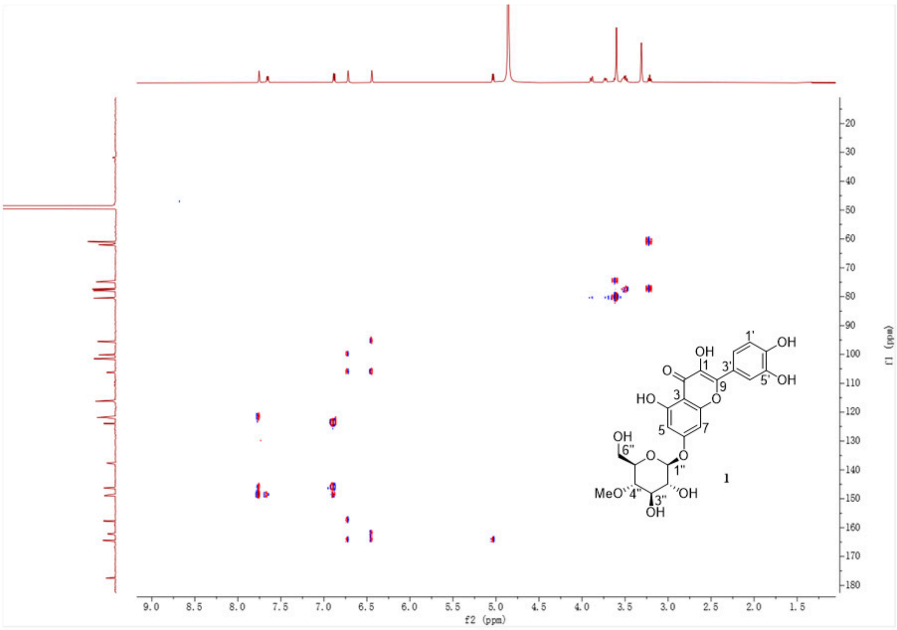

Figure S6. HMBC spectrum of compound **1** in MeOD

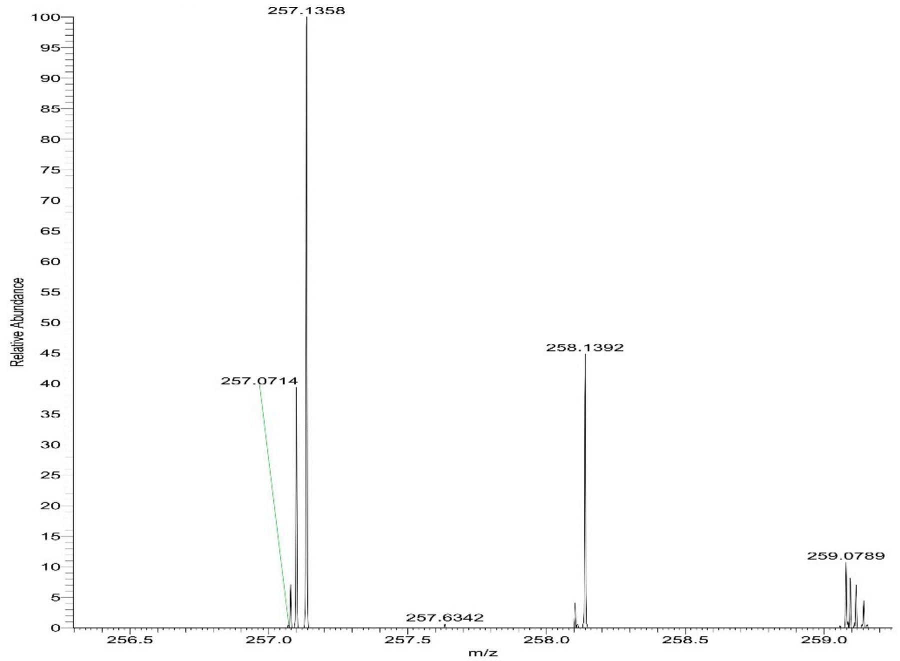

Figure S7. HRESIMS of compound **5.**

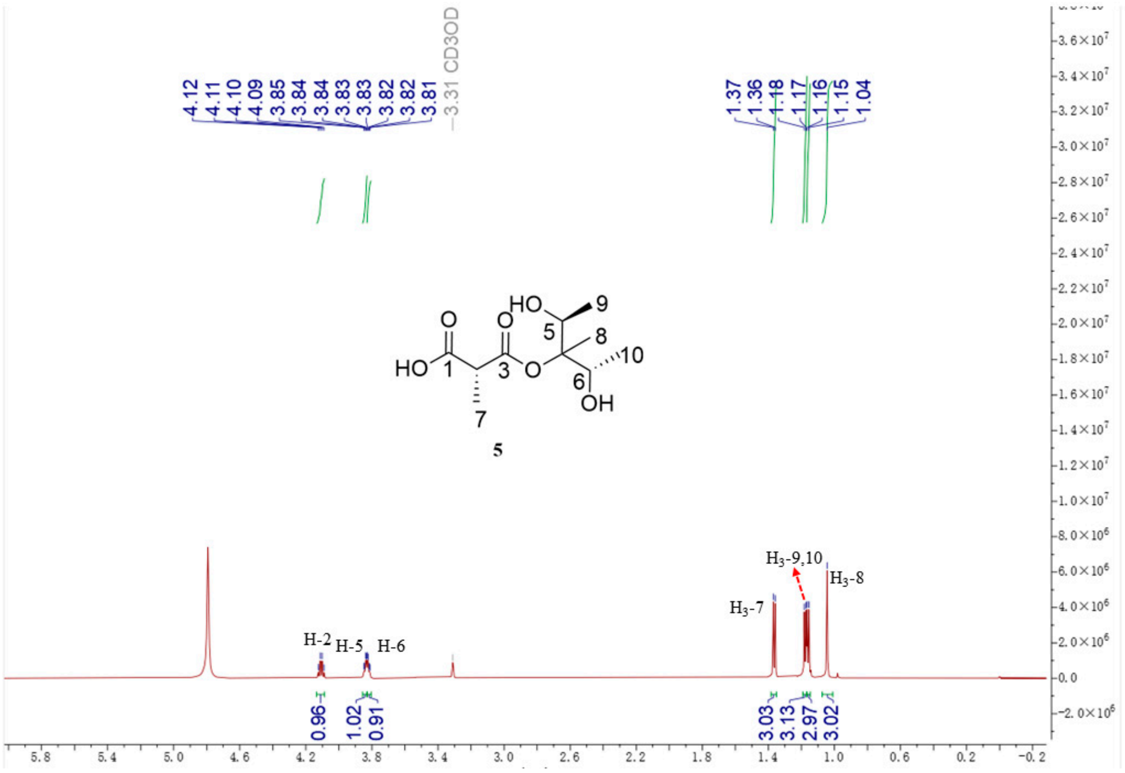

Figure S8. ^{1}H NMR spectrum of compound **5** in MeOD.

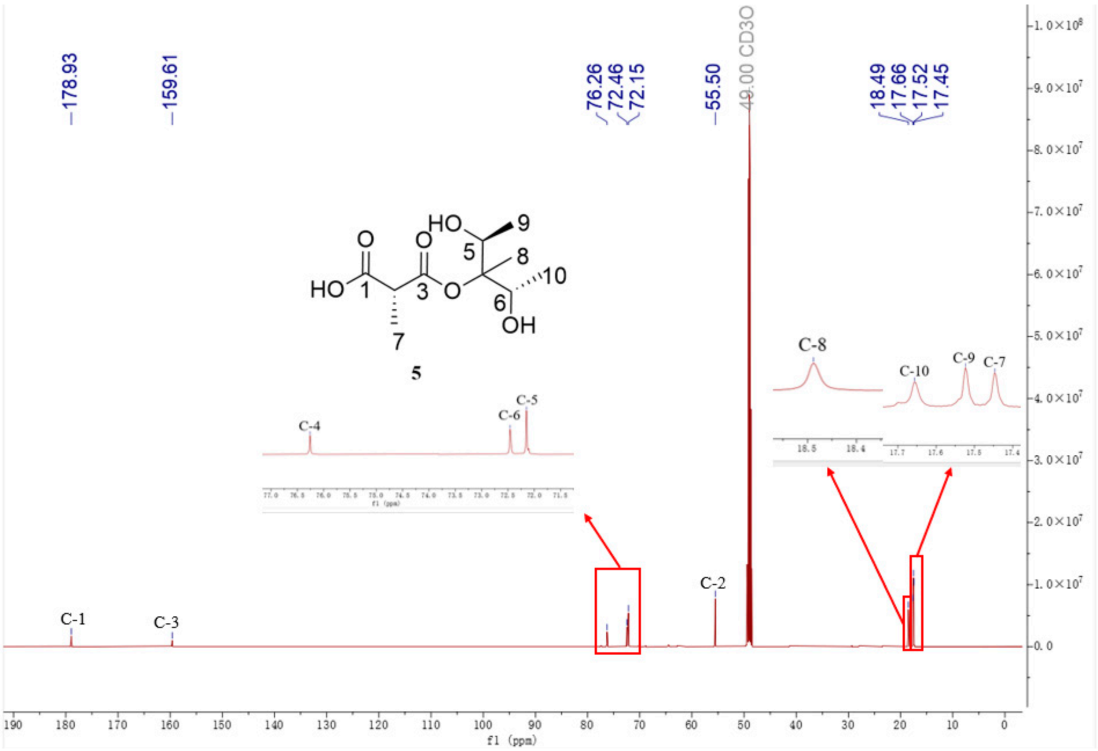

Figure S9. ^{13}C NMR spectrum of compound **5** in MeOD

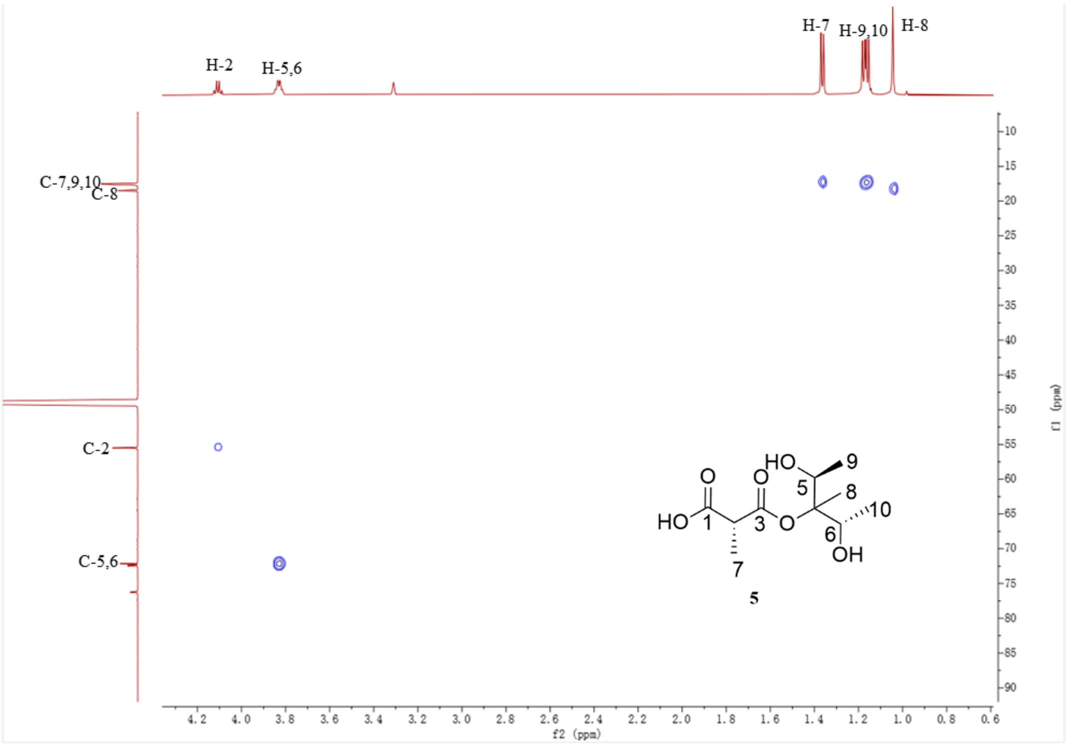

Figure S10. HSQC spectrum of compound **5** in MeOD.

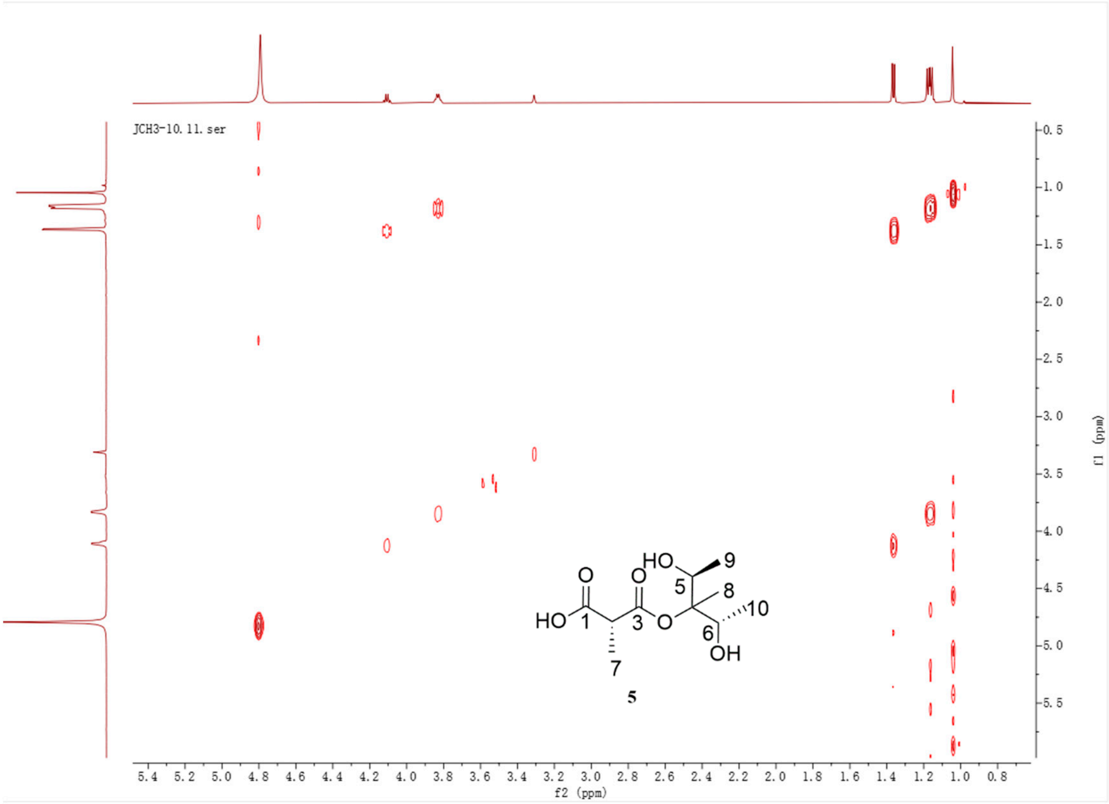

Figure S11. ¹H-¹H COSY spectrum of compound **5** in MeOD.

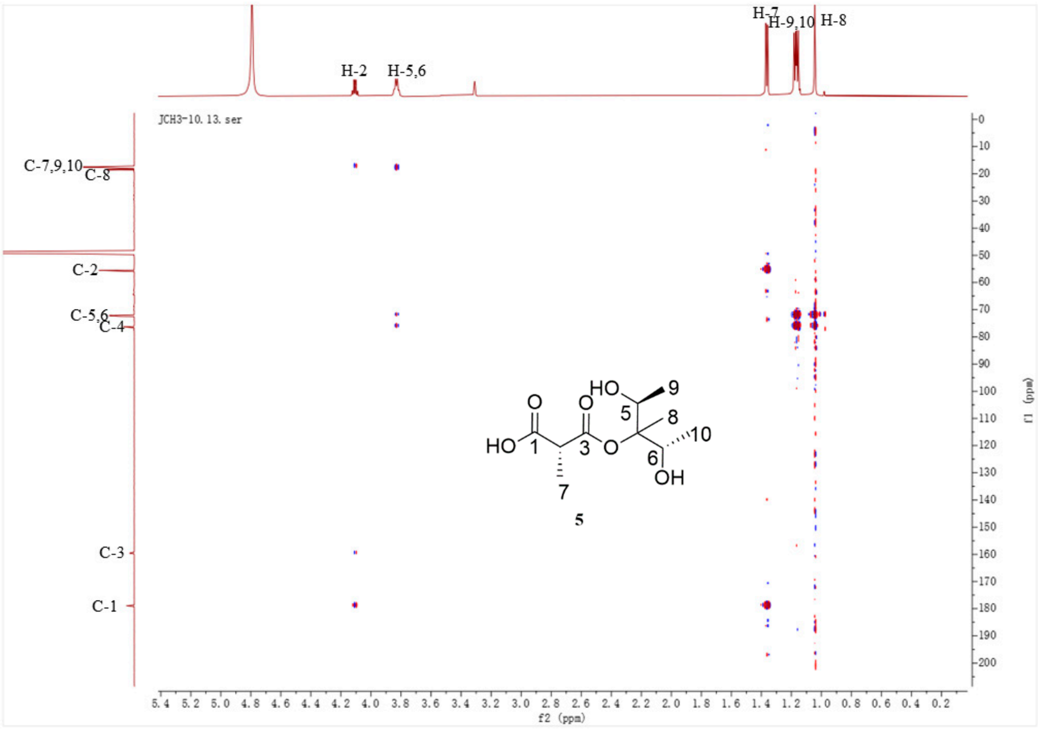

Figure S12. HMBC spectrum of compound **5** in MeOD.

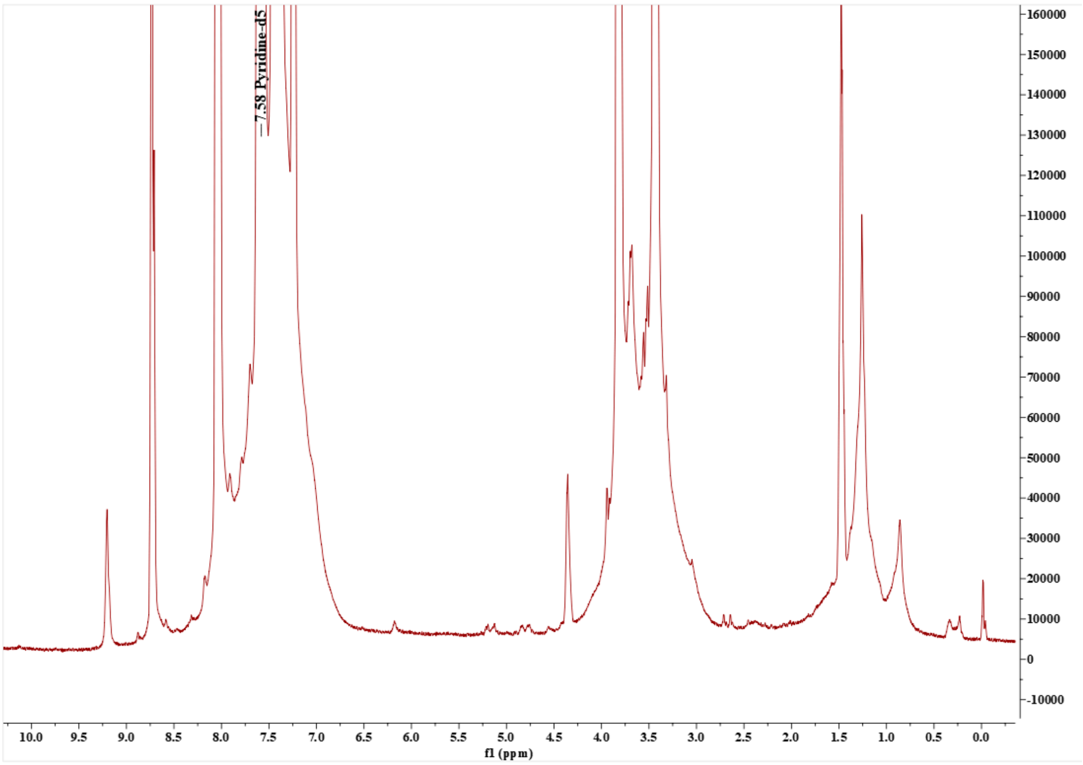

Figure S13. ^{1}H NMR spectrum of (*R*)-MTPA ester of **5**.

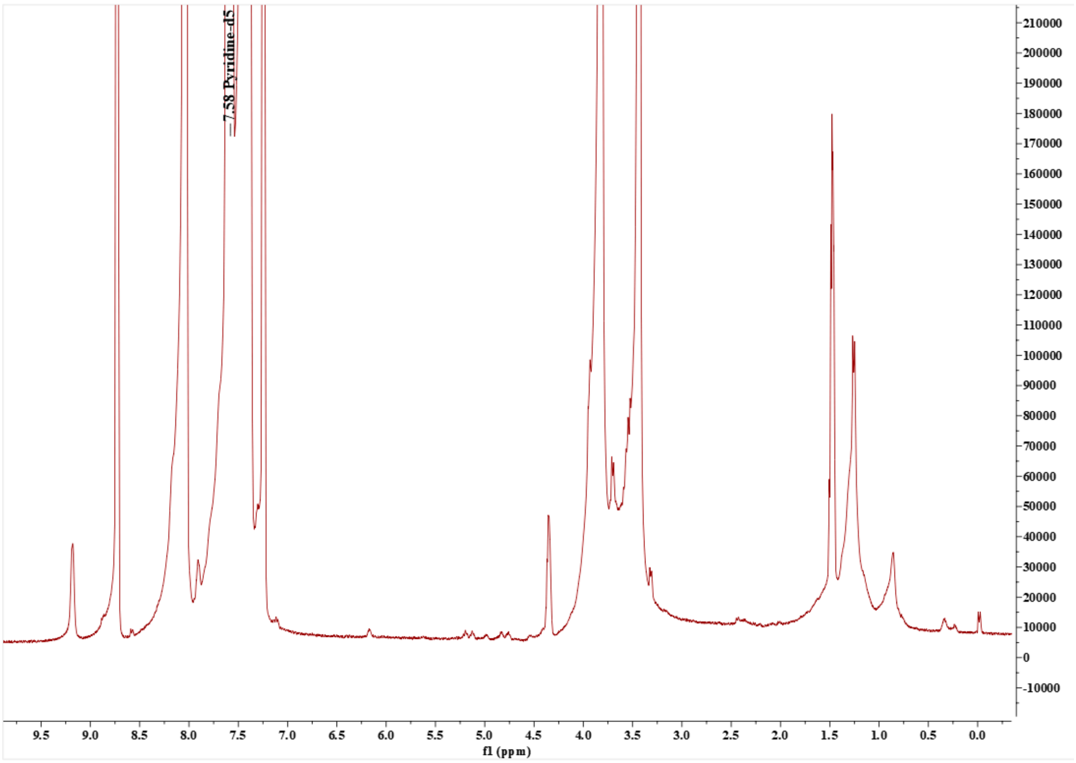

Figure S14. ^{1}H NMR spectrum of (*S*)-MTPA ester of **5**.

	2*R**5*R**6*R**	2*R**5*R**6*S**	2*R**5*S**6*R**	2*R**5*S**6*S**
	Isomer 1	Isomer 2	Isomer 3	Isomer 4
sDP4+ (H data)	5. 17%	11. 35%	11. 37%	72. 11%
sDP4+ (C data)	37. 01%	5. 71%	5. 74%	51. 55%
sDP4+ (all data)	4. 74%	1. 60%	1. 62%	92. 04%
uDP4+ (H data)	1. 69%	43. 02%	43. 11%	12. 18%
uDP4+ (C data)	14. 08%	10. 73%	6. 72%	68. 46%
uDP4+ (all data)	1. 48%	28. 68%	18. 01%	51. 83%
DP4+ (H data)	0. 47%	26. 16%	26. 28%	47. 09%
DP4+ (C data)	12. 56%	1. 48%	0. 93%	85. 04%
DP4+ (all data)	0. 14%	0. 95%	0. 60%	98. 31%

Figure S15. DP4+ analysis of compound 2*R**5*R**6*R**-, 2*R**5*R**6*S**-, 2*R**5*S**6*R**-, and 2*R**5*S**6*S**-**5**.

Known compounds 2-4 NMR and ESI-MS data:

Compound **2**: ^{1}H NMR (600 MHz, $CDCl_3$): δ_H 12.77 (s, 1H), 7.87 (s, 1H), 6.78 (s, 1H), 6.56 (s, 1H), 6.31(d, *J* = 2.1 Hz, 1H), 6.21 (d, *J* = 2.1 Hz, 1H), 3.78 (s, 3H), 3.66 (s, 3H) ^{13}C NMR (150 MHz, $CDCl_3$): δ_C 181.7, 162.8, 161.4, 157.6, 154.7, 152.9, 147.4, 140.7, 121.2, 114.4, 109.8, 105.4, 100.3, 99.1, 94.1, 56.3, 55.2 ESI-MS *m/z*: $[M + H]^+$ 331.08. Compound **2** was identified as 7-hydroxy-2′,4′,5′-trimethoxyisoflavone by comparison of its spectroscopic data with those reported in the literature [1].

Compound **3**: ^{1}H NMR (600 MHz, $CDCl_3$): δ_H 12.76 (s, 1H), 7.87(s, 1H), 6.96(d, *J*=2.2Hz, 1H), 6.88(s, 1H), 6.61(s, 1H), 6.35(d, *J*=2.2Hz, 1H), 3.91(s, 3H), 3.85(s, 3H), 3.84(s, 3H), 3.77(s, 3H) ^{13}C NMR (150 MHz, $CDCl_3$): δ_C 181.8, 164.5, 163.6, 157.9, 154.9, 152.5, 150.1, 143.1, 120.6, 115.1, 109.7, 105.3, 99.1, 98.1, 93.6, 56.8, 56.4, 56.2, 55.8 ESI-MS *m/z*: $[M + H]^+$ 359.11. Compound **3** was identified as olibergin A by comparison of its spectroscopic data with those reported in the literature [1].

Compound **4**: ^{1}H NMR (600 MHz, $CDCl_3$): δ_H 12.35 (s, 1H), 9.68 (s, 1H), 6.86 (s, 1H), 6.75 (s, 1H), 5.97 (d, *J*=2.0 Hz, 1H), 5.95 (d, *J*=2.0 Hz, 1H), 4.56 (dd, *J*=11.1, 12.0 Hz, 1H), 4.41 (dd, *J*=5.6, 11.1 Hz, 1H), 4.32 (dd, *J*=5.6, 12.0 Hz, 1H), 3.84 (s, 3H), 3.80 (s, 3H), 3.72 (s, 3H); ^{13}C NMR (150 MHz, $CDCl_3$): δ_C 197.2, 165.9, 164.8, 163.6, 152.4, 151.6, 145.0, 117.3, 116.0, 104.2, 101.4, 97.4, 96.6, 72.2, 57.3, 57.0, 56.8, 48.3 ESI-MS *m/z*: $[M + H]^+$ 347.11. Compound **4** was identified as (2*R*)-2,3-dihydro-7-demethylrobustigenin by comparison of its spectroscopic data with those reported in the literature [2].

References

(1) Arthan, S.; Posri, P.; Walunchapruk, S.; Senawong, T.; Yenjai, C. Structural Modification of Olibergin A, an Isoflavonoid, from *Dalbergia Stipulacea* Roxb. and Its Cytotoxicity. *RSC Adv.* 2022, *12*, 17837–17845. https://doi.org/10.1039/D2RA02865D.

(2) Zarev, Y.; Foubert, K.; Lucia De Almeida, V.; Anthonissen, R.; Elgorashi, E.; Apers, S.; Ionkova, I.; Verschaeve, L.; Pieters, L. Antigenotoxic Prenylated Flavonoids from Stem Bark of Erythrina Latissima. *Phytochemistry* 2017, *141*, 140–146. https://doi.org/10.1016/j.phytochem.2017.06.003.
